# TMEM41A overexpression correlates with poor prognosis and immune alterations in patients with endometrial carcinoma

**DOI:** 10.1371/journal.pone.0285817

**Published:** 2023-07-21

**Authors:** Ke Shi, Xiao-Li Liu, Qiang Guo, Yun-Qiang Zhang, Si-Tong Fan, Ling Dai, Ni Jiang, Dan Li

**Affiliations:** 1 Department of Thoracic Surgery, Beilun District People’s Hospital of Ningbo, Ningbo City, China; 2 Department of Ultrasound, The People’s Hospital of Jianyang City, Jianyang City, China; 3 Department of Cardiothoracic Surgery, Taihe Hospital, Hubei Medical University, Shiyan City, China; 4 Department of Infectious Disease, Beilun District People’s Hospital of Ningbo, Ningbo City, China; 5 Department of Obstetrics and Gynecology, Women and Children’s Hospital of Chongqing Medical University, Chongqing City, China; 6 Department of Oncology, Taihe Hospital, Hubei Medical University, Shiyan City, China; Sapienza University of Rome: Universita degli Studi di Roma La Sapienza, ITALY

## Abstract

**Background:**

Expression levels of transmembrane protein 41A (*TMEM41A)* are related to the progression of malignant tumors. However, the association between *TMEM41A* expression and endometrial carcinoma (EC) remains unclear. This study aims to identify the roles of *TMEM41A* expression in the prognosis of patients with EC and its correlation with EC progression.

**Methods:**

The *TMEM41A* expression and its correlation with the survival of patients with EC were assessed. Cox regression analysis was used to identify the prognostic factors, while nomograms were used to examine the association between the prognostic factors and the survival of patients with EC. Finally, the link between *TMEM41A* level and immune microenvironment and RNA modifications was investigated in EC.

**Results:**

*TMEM41A* was overexpressed in EC. *TMEM41A* overexpression could diagnose the EC and evaluate the poor prognosis of patients. Overexpression of *TMEM41A* was associated with clinical stage, age, weight, histological subtype, tumor grade, and survival status of patients with EC. Clinical stage, age, tumor grade, radiotherapy, and *TMEM41A* overexpression were factors of poor prognosis in patients with EC. The nomograms revealed the correlation between the *TMEM41A* level and survival time of patients with EC at 1, 3, and 5 years. Furthermore, *TMEM41A* overexpression was significantly correlated with the level of the stromal score, immune score, estimate score, NK CD56 bright cells, iDC, NK cells, eosinophils, pDC, T cells, TReg, cytotoxic cells, mast cells, Th17 cells, neutrophils, aDC, NK CD56 dim cells, TFH, Th2 cells, CD8 T cells, macrophages, immune cell markers, and RNA modifications.

**Conclusions:**

*TMEM41A* is overexpressed in EC tissues and is associated with the prognosis, immune microenvironment, and RNA modification. Our preliminary studies indicate that overexpression of *TMEM41A* can potentially serve as a biomarker for EC treatment.

## 1. Introduction

Endometrial carcinoma (EC) is a common type of malignant epithelial tumor in women, with 10%-15% of patients with EC being diagnosed at an advanced stage, leading to unfavorable prognosis [1, 2]. While surgery and adjuvant treatment can improve the survival time of early patients with EC, there is no effective treatment for those with advanced cancer [1]. Over the years, several genes with crucial biological functions have emerged as research hotspots [3–5]. For instance, the combination of PARP inhibitor and PD-1/PD-L1 checkpoint inhibitor could improve the survival time of patients with cancer [3]. Therefore, identifying novel therapeutic targets and early diagnostic markers is critical in cancer.

Recent studies have shown the association between transmembrane protein 41A (*TMEM41A)* and the occurrences and developments of gastrointestinal tumors [6, 7], with significantly higher expression levels of SREBF pathway regulator in golgi 1 (*SPRING1*) in colorectal cancer than in adjacent normal tissues. *In vitro* and *in vivo* studies found that *SPRING1* could enhance the growth of colorectal cancer cells, which was related to the increased expression of *TMEM41A* [6]. *TMEM41A* was strongly expressed in gastric cancer and linked to lymph node metastasis, distant metastasis, late stage, and poor prognosis. Inhibition of *TMEM41A* expression could reduce gastric cancer cell migration and metastasis by inhibiting the epithelial-mesenchymal transformation (EMT) process and autophagy [7]. Collectively, these preliminary findings indicate that *TMEM41A* is crucial in promoting cell growth and migration as an oncogene.

The Cancer Genome Atlas (TCGA) database provides transcriptome data of normal and cancer tissues of patients with cancer [8–10], which has been extensively analyzed in various studies [11–13]. For example, the down-regulation of B cell translocation gene 1 (*BTG1*) expression was related to poor prognosis, degree of invasion, and FIGO stage in patients with EC. Overexpression of *BTG1* inhibits proliferation, migration, and invasion as well as promotes apoptosis of EC cells, which was related to the EMT process [13]. The correlation between *TMEM41A* expression and EC remains unclear. Therefore, this study aims to comprehensively analyze the roles and possible mechanisms of *TMEM41A* in EC and to identify potential candidate molecules for cancer treatment.

## 2. Materials and methods

### 2.1. Data acquisition and identification of *TMEM41A* expression

The transcriptome data of patients with cancer were obtained from the TCGA (https://portal.gdc.cancer.gov/repository) database in the FPKM file format. Among them, 35 cases were normal tissue, and 554 were cancer tissue samples. Additionally, 23 normal tissue and 23 cancer tissue samples were paired. The expression levels of *TMEM41A* in EC were analyzed by expression analysis.

### 2.2. Diagnostic evaluation and prognostic analysis of *TMEM41A*

Receiver operating characteristic (ROC) analysis on TCGA FPKM data to determine the area under the curve (AUC) and identify its diagnostic value in normal and EC tissues. The data of *TMEM41A* expression was combined with the prognosis data, and the missing data were excluded. After classifying the samples according to the median expression of *TMEM41A*, the association between changes in *TMEM41A* expression and the poor prognosis of patients with EC was identified using survival analysis.

### 2.3. Clinical characteristics analysis

The clinical characteristics of patients with EC were obtained from the TCGA database. These data included the clinical stage, age, weight, histological subtype, tumor grade, and survival status, which were used to group the samples, and *TMEM41A* expression in these groups was analyzed. Moreover, the median expression of *TMEM41A* was used to divide the samples into high- and low- expression groups to explore the correlation between *TMEM41A* levels and clinical characteristics of patients with EC.

### 2.4. Survival analysis in subgroups of patients with cancer

The clinical stage (I, II, III, and IV), weight, height, BMI, and tumor grade of patients with EC were analyzed. The patients were divided into high- and low-expression groups to explore the link between *TMEM41A* levels and overall survival (OS), disease-specific survival (DSS), and disease progression in patients with EC.

### 2.5. Establishment of *TMEM41A*-related nomograms based on the Cox method

The clinical data of patients with EC patients and *TMEM41A* expression in cancer tissues from the TCGA database were matched. The risk indicators of poor prognosis were filtered using the univariate Cox regression analysis with *P* < 0.05 as the screening criteria. Subsequently, multivariate Cox regression analysis was performed, and its results were used to construct the *TMEM41A*-related nomograms.

### 2.6. Analysis of immune microenvironment

Single-sample gene set enrichment analysis (ssGSEA) and ESTIMATE algorithms were used to evaluate the levels of immune microenvironment components in EC tissues. Correlation analysis was used to investigate the association between *TMEM41A* levels and stromal score, dendritic cell (DC), Tcm, immune score, Tgd, B cells, estimate score, Th1 cells, T helper cells, Tem, CD8 T cells, NK CD56dim cells, TFH, Th2 cells, macrophages, cytotoxic cells, Th17 cells, neutrophils, aDC, T cells, TReg, mast cells, eosinophils, pDC, NK cells, iDC, and NK CD56bright cells.

### 2.7. Correlation between *TMEM41A* expression and immune cell markers

The expression data of immune cell markers and *TMEM41A* in 554 EC tissues were obtained, and their correlation was investigated using the Spearman correlation analysis.

### 2.8. TIMER database

The TIMER (https://cistrome.shinyapps.io/timer) database was a cancer immune-related database. The relationship between *TMEM41A* expression and cancer immune cells was analyzed in the gene module of the TIMER database, and the link between *TMEM41A* expression and cancer immune cell copy number was analyzed in the somatic copy number alteration (SCNA) module of the TIMER database.

### 2.9. Association between the expression levels of *TMEM41A* and RNA modification

The *TMEM41A* was input into the RM2Target online database to obtain the *TMEM41A*-related RNA modification genes, which were restricted according to the artificial filtering standard of species. The expression data of RNA modification genes and *TMEM41A* in 554 EC tissues were obtained, and their association was investigated using Spearman correlation analysis.

### 2.10. Statistical analysis

Wilcoxon rank sum test and chi-square test were used to detect the expression levels of *TMEM41A* in EC. The ROC and survival analyses examined the link between *TMEM41A* expression and EC diagnosis and patient survival time. Spearman correlation analysis assessed the correlation between *TMEM41A* expression and immune microenvironment and RNA modification genes. A *P-*value < 0.05 was considered statistically significant.

## 3. Results

### 3.1. Increased expression of *TMEM41A* is valuable in the diagnosis and prediction of poor prognosis in patients with EC

**Fig 1:**
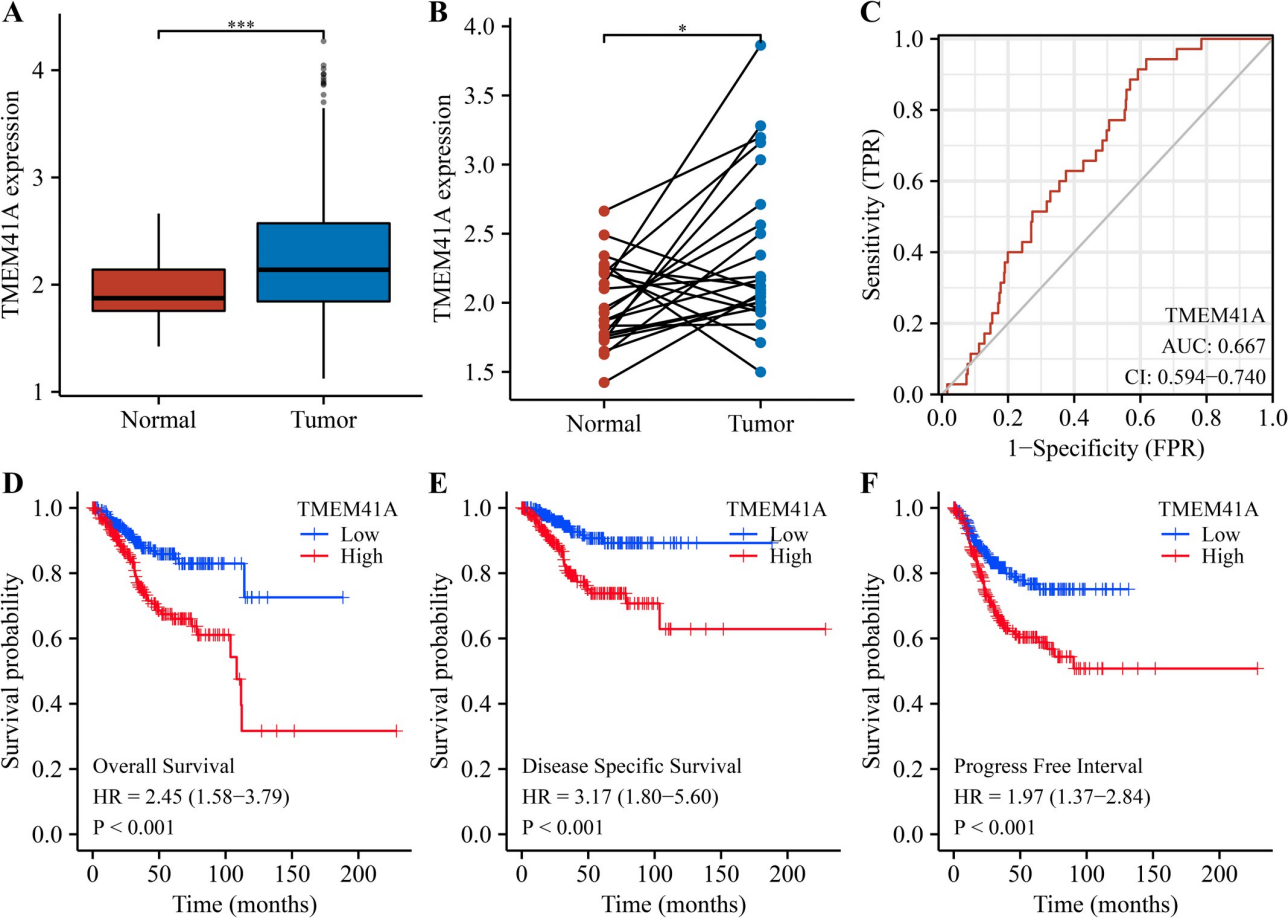
Increased *TMEM41A* expression correlates to the diagnosis and poor prognosis in EC. (A) *TMEM41A* expression in unpaired tissues. (B) *TMEM41A* expression in paired tissues. (C) The area under the curve of *TMEM41A* in EC. (D-F) *TMEM41A* overexpression correlates to the poor prognosis in EC. EC, endometrial cancer.

The expression analysis based on the TCGA data revealed that *TMEM41A* was overexpressed both in non-paired (Fig 1A) and paired EC tissues (Fig 1B). Furthermore, ROC analysis demonstrated that the area under the curve of *TMEM41A* was 0.667, indicating that *TMEM41A* had a diagnostic value for EC (Fig 1C). Finally, survival analysis showed that enhanced *TMEM41A* expression was associated with shorter OS, DSS, and progression-free interval (PFI) among patients with EC (Fig 1D–1F).

### 3.2. *TMEM41A* level correlates with clinicopathological features of patients with EC

**Fig 2:**
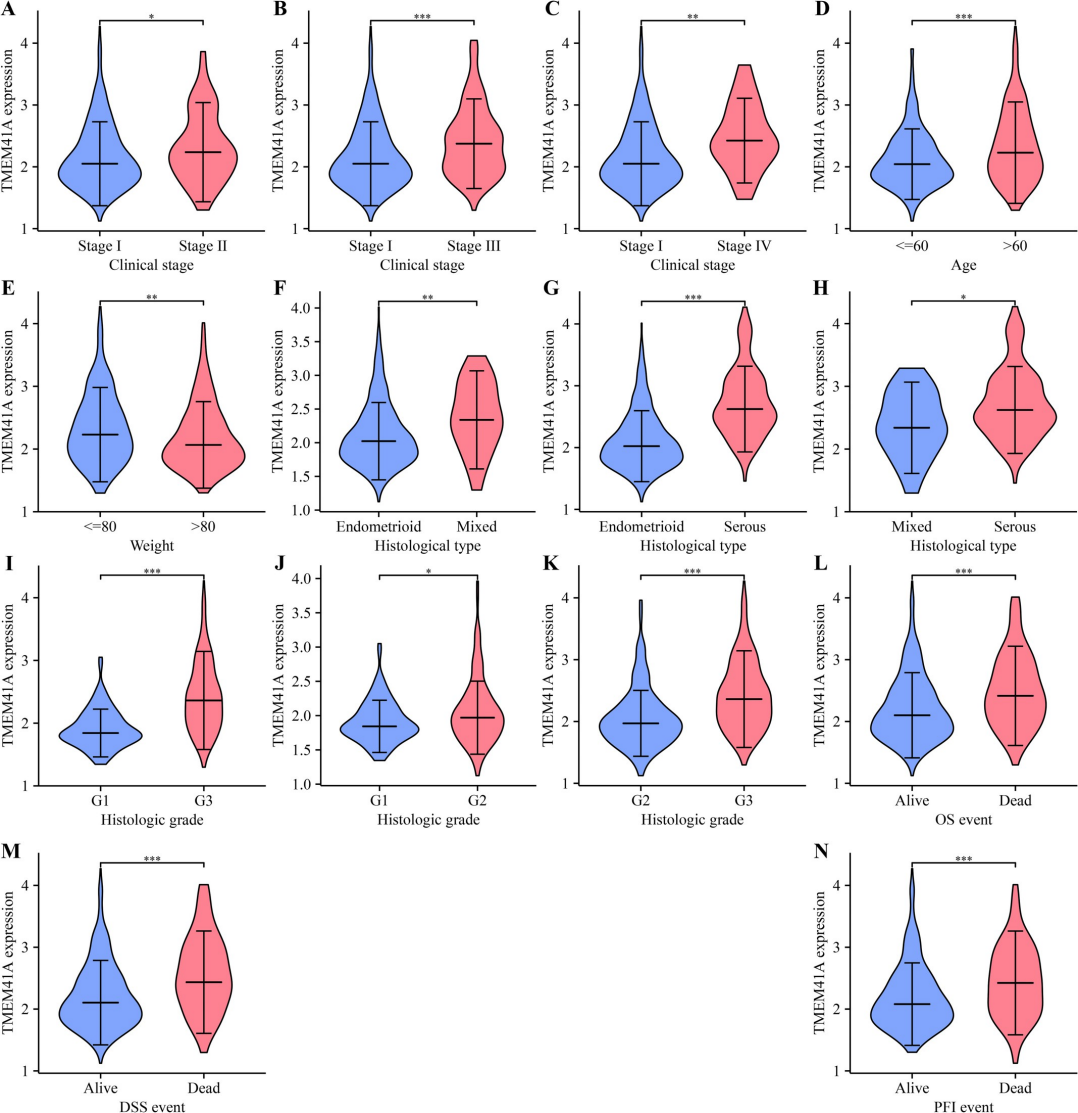
*TMEM41A* expression correlates with the clinical indicators in EC. (A-N) Correlation analysis between *TMEM41A* expression and clinical stage (A-C), age (D), weight (E); histological type (F-H), histologic grade (I-K), OS event (L), DSS event (M), and PFI event (N). EC, endometrial cancer; OS, overall survival; DSS, disease-specific survival; PFI, progression-free interval.

Our results highlighted a significant correlation between *TMEM41A* expression and various clinicopathological features in patients with EC (Fig 2). Specifically, *TMEM41A* was strongly expressed in cancer tissues from patients with clinical stage II, III, and IV EC compared with those of clinical stage I (Fig 2A–2C). Furthermore, *TMEM41A* was strongly expressed in cancer tissues of patients above 60 (Fig 2D) and those with a weight greater than 80 kg (Fig 2E). Moreover, *TMEM41A* was strongly expressed in mixed and serous subtype patients, compared with endometrioid subtype. In addition, *TMEM41A* was strongly expressed in serous patient carcinoma tissues compared to patients with mixed subtypes (Fig 2F–2H). *TMEM41A* was strongly expressed in cancer tissues of patients with G2 and G3 stages compared with those of G1 stage and in cancer tissues of G3 patients compared with the G2 patients (Fig 2I–2K). Notably, *TMEM41A* expression levels were significantly enhanced in cancer tissues from deceased patients compared to those from living patients (Fig 2L–2N). Statistical analyses revealed that high- and low-*TMEM41A* expression was associated with clinical stage, PFI, race, age, DSS, weight, OS, histologic type, histologic grade, and menopausal status in patients with EC (Table 1).

**Table 1:** *TMEM41A* expression associates with clinical indicators in patients with EC.

Characteristic	Low TMEM41A expression	High TMEM41A expression	P
N	276	276	
Clinical stage			< 0.001
Stage I	197 (35.7%)	145 (26.3%)	
Stage II	21 (3.8%)	30 (5.4%)	
Stage III	52 (9.4%)	78 (14.1%)	
Stage IV	6 (1.1%)	23 (4.2%)	
Race			0.029
Asian	13 (2.6%)	7 (1.4%)	
Black or African American	44 (8.7%)	64 (12.6%)	
White	203 (40%)	176 (34.7%)	
Age			< 0.001
< = 60	125 (22.8%)	81 (14.8%)	
>60	151 (27.5%)	192 (35%)	
Weight			0.001
< = 80	103 (19.5%)	140 (26.5%)	
>80	162 (30.7%)	123 (23.3%)	
Height			0.369
< = 160	120 (22.9%)	127 (24.3%)	
>160	146 (27.9%)	130 (24.9%)	
BMI			0.095
< = 30	98 (18.9%)	114 (22%)	
>30	166 (32%)	141 (27.2%)	
Histological type			< 0.001
Endometrioid	254 (46%)	156 (28.3%)	
Mixed	7 (1.3%)	17 (3.1%)	
Serous	15 (2.7%)	103 (18.7%)	
Histologic grade			< 0.001
G1	79 (14.6%)	19 (3.5%)	
G2	83 (15.3%)	37 (6.8%)	
G3	111 (20.5%)	212 (39.2%)	
Tumor invasion (%)			0.412
<50	141 (29.7%)	118 (24.9%)	
> = 50	108 (22.8%)	107 (22.6%)	
Menopause status			0.011
Pre	24 (4.7%)	11 (2.2%)	
Peri	12 (2.4%)	5 (1%)	
Post	215 (42.5%)	239 (47.2%)	
Hormones therapy			0.776
No	154 (44.8%)	143 (41.6%)	
Yes	26 (7.6%)	21 (6.1%)	
Diabetes			0.870
No	166 (36.8%)	162 (35.9%)	
Yes	64 (14.2%)	59 (13.1%)	
Radiation therapy			0.067
No	155 (29.4%)	124 (23.5%)	
Yes	117 (22.2%)	131 (24.9%)	
OS event			< 0.001
Alive	247 (44.7%)	211 (38.2%)	
Dead	29 (5.3%)	65 (11.8%)	
DSS event			< 0.001
Alive	260 (47.3%)	227 (41.3%)	
Dead	16 (2.9%)	47 (8.5%)	
PFI event			< 0.001
Alive	231 (41.8%)	192 (34.8%)		
Dead	45 (8.2%)	84 (15.2%)		

EC, endometrial cancer.

### 3.3. *TMEM41A* overexpression associates with the poor prognosis of patients with EC in subgroup analysis

**Fig 3:**
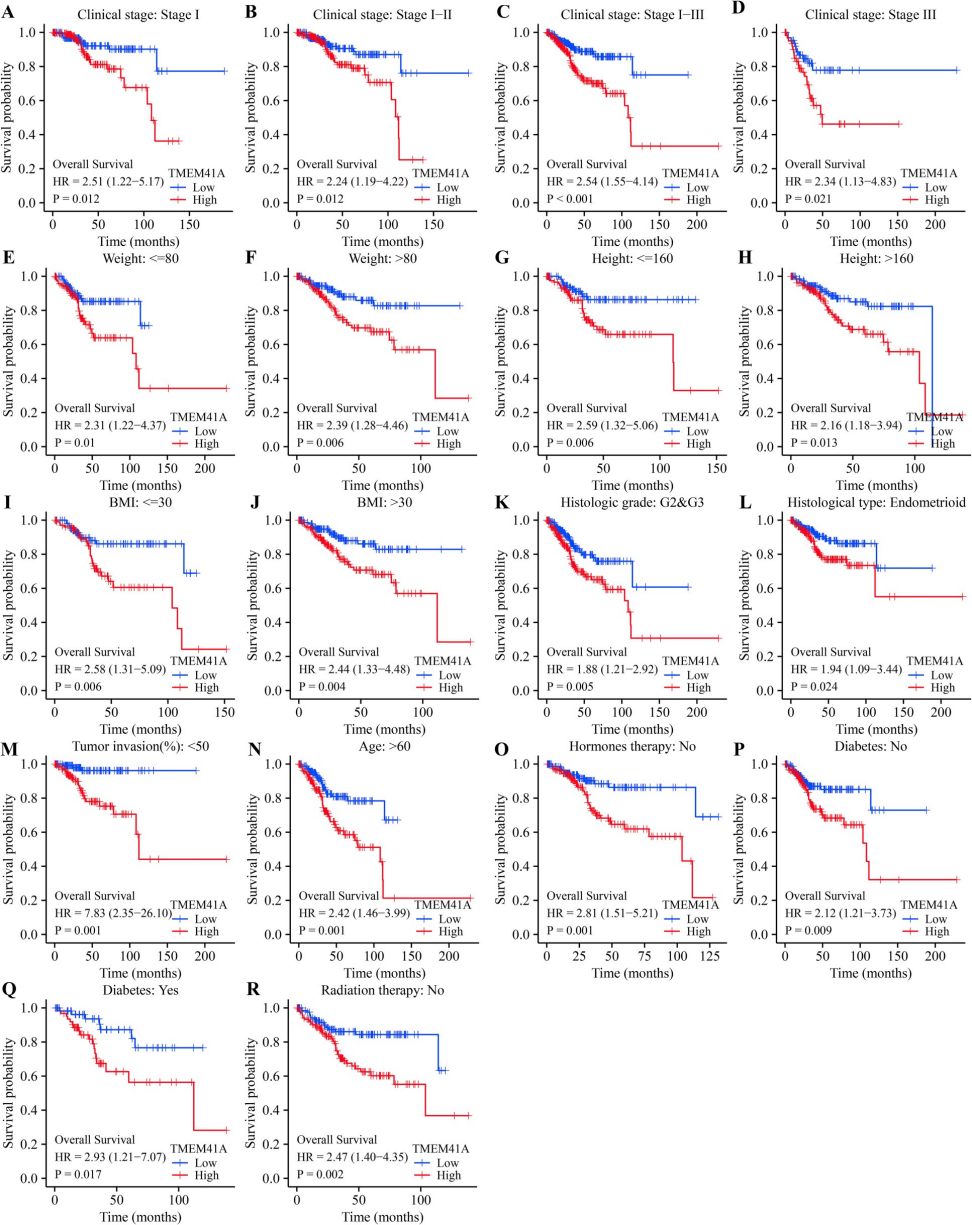
Overexpression of *TMEM41A* associates with poor OS in EC. EC, endometrial cancer; OS, overall survival.

**Fig 4:**
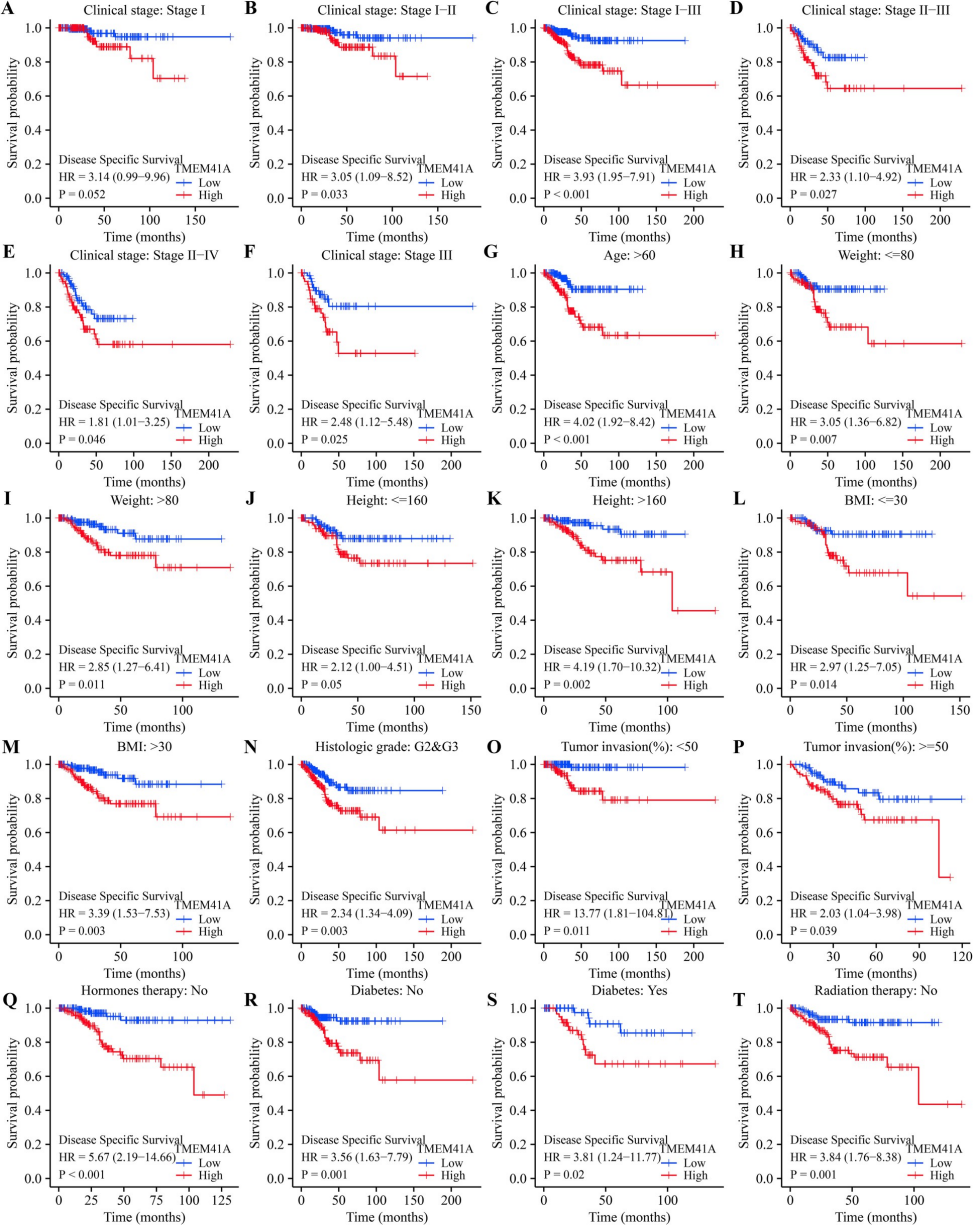
Overexpression of *TMEM41A* associates with poor DSS in EC. EC, endometrial cancer; DSS, disease-specific survival.

**Fig 5:**
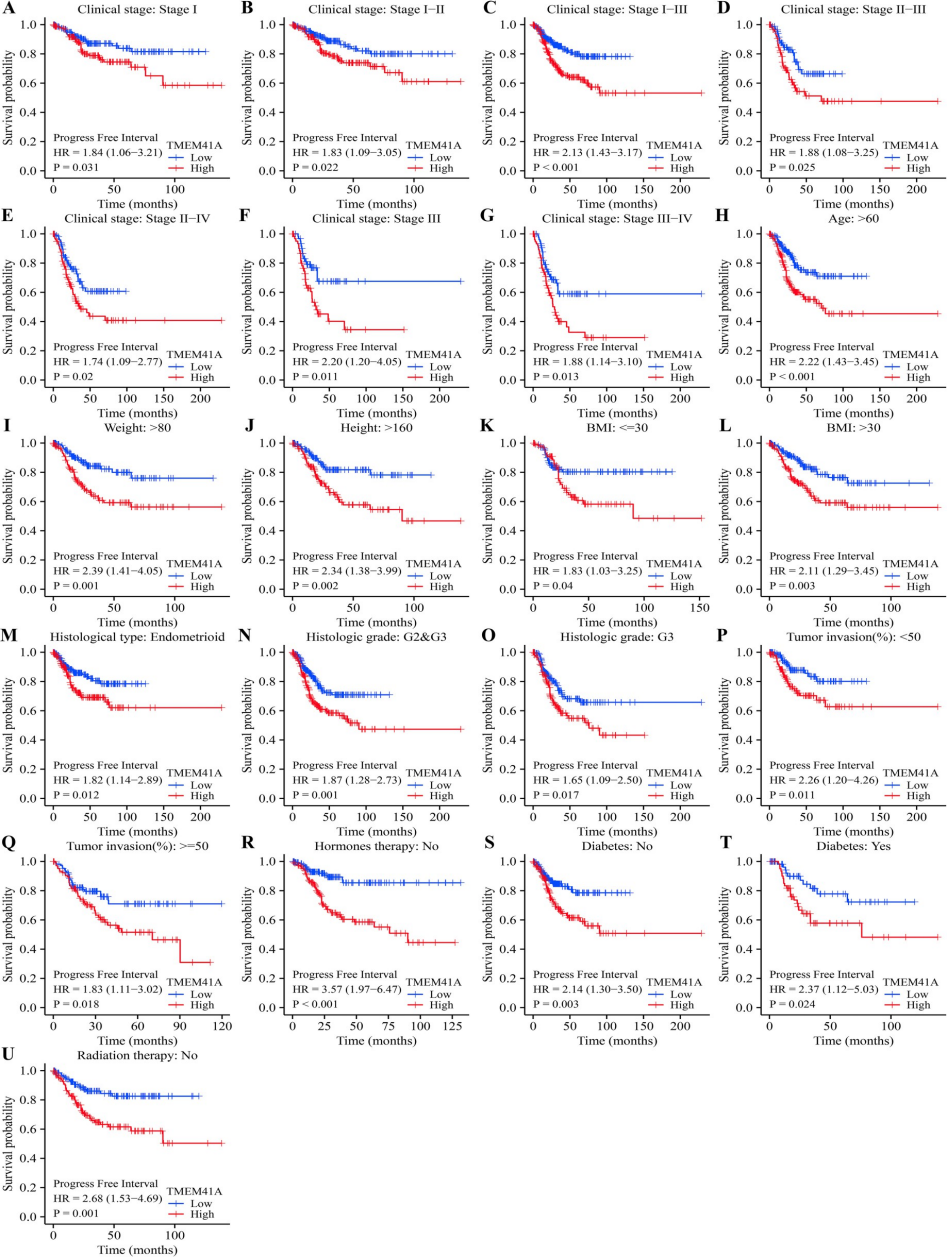
Overexpression of *TMEM41A* associates with poor PFI in EC. EC, endometrial cancer; PFI, progression-free interval.

The overexpression of *TMEM41A* was significantly associated with poor survival and progression in patients with EC grouped by clinical stage, age, weight, and others (Figs 3–5). Specifically, *TMEM41A* overexpression was associated with shorter OS (Fig 3) in patients with EC with stages I, I-II, I-III, and III, body weight ≤ 80kg, or > 80kg, height, BMI, G2-3 grade, endometrioid, tumor invasion, age ≥ 60 years, hormone therapy (no), radiotherapy (no), diabetes (yes or no). Similarly, *TMEM41A* overexpression was associated with shorter DSS in patients with EC with stages I, I-II, I-III, II-III, II-IV, and III, weight ≤ 80 or > 80kg, height, BMI, G2-3 grade, endometrioid, tumor invasion, age > 60 years, hormone therapy (no), radiotherapy (no), diabetes (yes or no) (Fig 4). Furthermore, *TMEM41A* overexpression was associated with shorter PFI in EC patients with stages I, I-II, I-III, II-III, II-IV, III, and III-IV, weight > 80kg, height > 160cm, BMI, G2-3 grade, G3 grade, endometrioid, tumor invasion, age > 60 years, hormone therapy (no), radiotherapy (no), and diabetes (yes or no) (Fig 5).

### 3.4. Construction of *TMEM41A*-related nomograms

**Fig 6:**
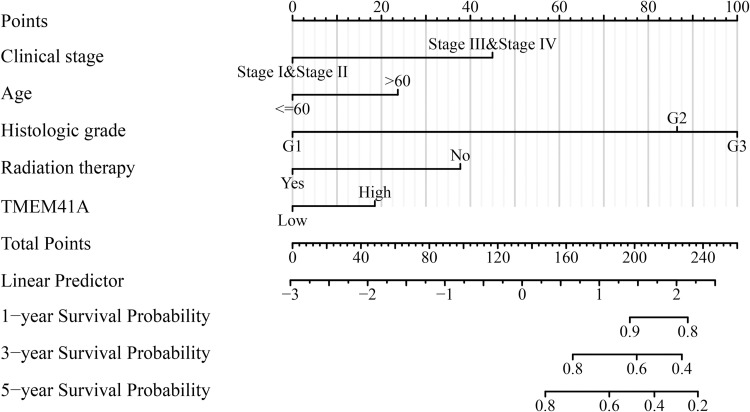
*TMEM41A*-related nomogram in OS of EC patients. EC, endometrial cancer; OS, overall survival.

**Fig 7:**
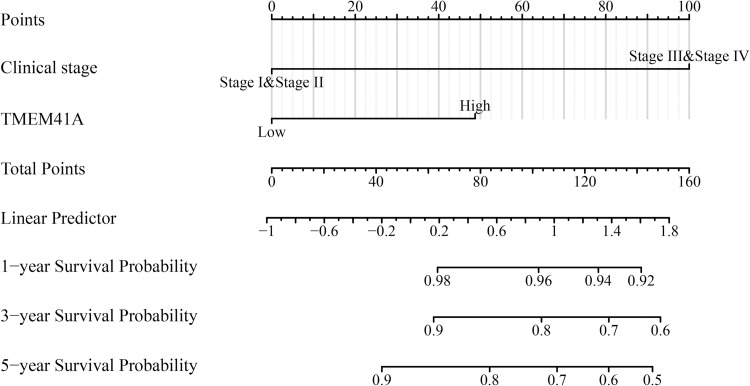
*TMEM41A*-related nomogram in DSS of EC patients. EC, endometrial cancer; DSS, disease-specific survival.

**Fig 8:**
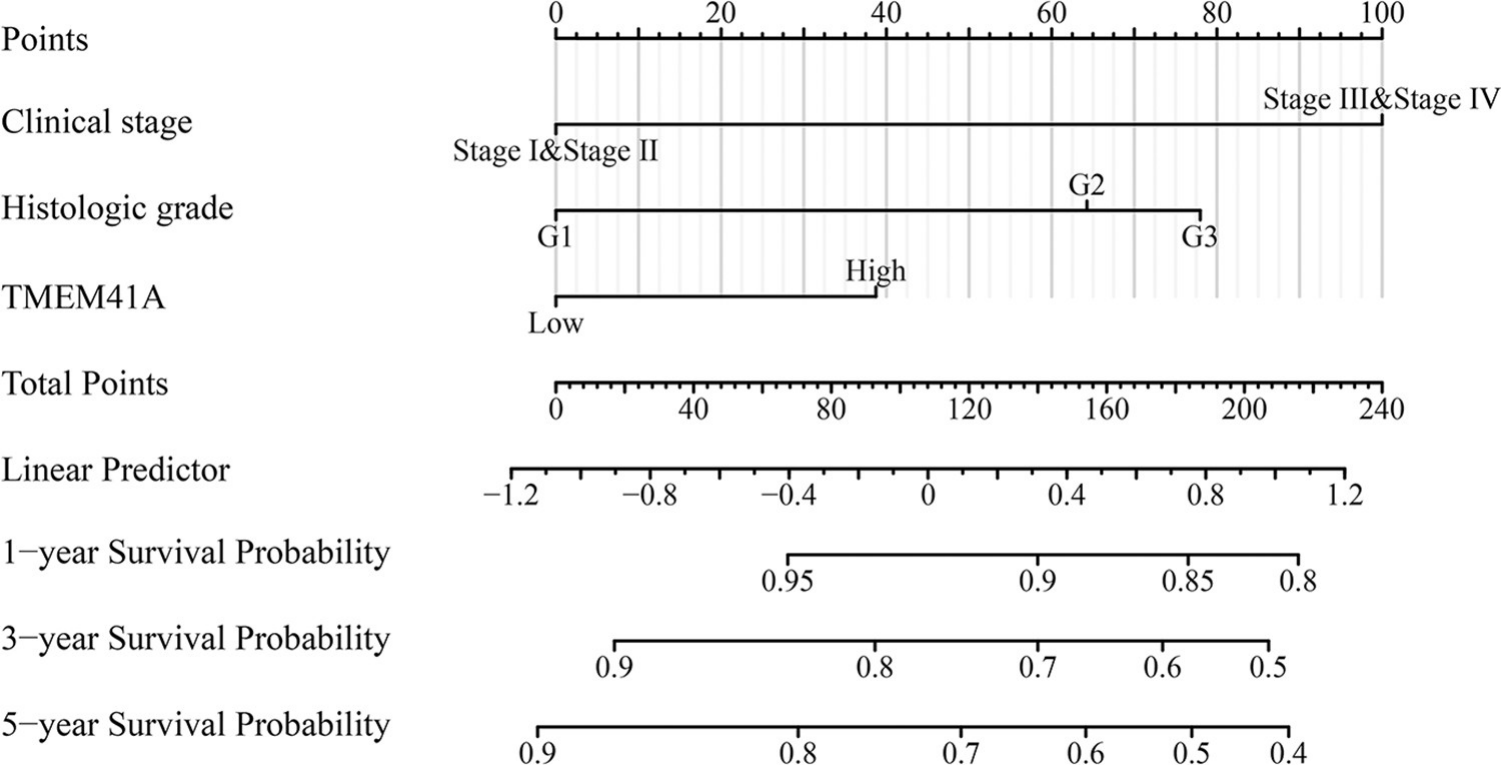
*TMEM41A*-related nomogram in PFI of EC patients. EC, endometrial cancer; PFI, progression-free interval.

Univariate Cox regression analysis revealed that clinical stage, age, histological grade, radiotherapy, and *TMEM41A* overexpression were risk factors for shorter OS in patients with EC (Table 2). Similarly, clinical stage and *TMEM41A* overexpression were risk factors for shorter DSS in patients with EC (Table 3). Moreover, clinical stage, histologic grade, and *TMEM41A* overexpression were risk factors for shorter PFI in patients with EC (Table 4). We further showed the association between adverse factors in patients with EC and patient prognosis to predict patient survival time via nomogram (Figs 6–8).

**Table 2:** Factors associated with poor OS in EC.

Characteristics	Total (N)	HR (95% CI)	P
Clinical stage	551		
Stage I	341	Reference	
Stage II	51	1.751 (0.840–3.653)	0.135
Stage III	130	3.078 (1.907–4.968)	<0.001
Stage IV	29	8.065 (4.488–14.495)	<0.001
Age	549		
< = 60	206	Reference	
>60	343	1.847 (1.160–2.940)	0.010
Weight	527		
< = 80	242	Reference	
>80	285	1.060 (0.699–1.607)	0.784
Height	522		
< = 160	246	Reference	
>160	276	1.153 (0.758–1.753)	0.507
BMI	518		
< = 30	211	Reference	
>30	307	0.967 (0.636–1.470)	0.876
Histologic grade	540		
G1	98	Reference	
G2	120	7.117 (1.617–31.326)	0.009
G3	322	13.241 (3.247–53.993)	<0.001
Radiation therapy	527		
No	279	Reference	
Yes	248	0.594 (0.385–0.915)	0.018
TMEM41A	551		
Low	276	Reference	
High	275	2.446 (1.578–3.792)	<0.001

EC, endometrial cancer; OS, overall survival.

**Table 3:** Factors associated with poor DSS in EC.

Characteristics	Total (N)	HR (95% CI)	P
Clinical stage	549		
Stage I	340	Reference	
Stage II	50	1.785 (0.592–5.382)	0.304
Stage III	130	5.935 (3.160–11.145)	<0.001
Stage IV	29	16.815 (8.274–34.173)	<0.001
Age	547		
< = 60	206	Reference	
>60	341	1.215 (0.724–2.042)	0.461
Weight	525		
< = 80	241	Reference	
>80	284	0.912 (0.551–1.510)	0.721
Height	520		
< = 160	244	Reference	
>160	276	0.886 (0.533–1.472)	0.640
BMI	516		
< = 30	210	Reference	
>30	306	0.948 (0.569–1.581)	0.839
Histologic grade	538		
G1	98	Reference	
G2	120	34365084.838 (0.000-Inf)	0.995
G3	320	134915449.923 (0.000-Inf)	0.994
Radiation therapy	525		
No	277	Reference	
Yes	248	0.599 (0.351–1.021)	0.060
TMEM41A	549		
Low	276	Reference	
High	273	3.174 (1.799–5.599)	<0.001

EC, endometrial cancer; DSS, disease-specific survival.

**Table 4:** Factors associated with poor PFI in EC.

Characteristics	Total (N)	HR (95% CI)	P
Clinical stage	551		
Stage I	341	Reference	
Stage II	51	1.016 (0.502–2.058)	0.965
Stage III	130	2.581 (1.740–3.827)	<0.001
Stage IV	29	6.832 (4.081–11.437)	<0.001
Age	549		
< = 60	206	Reference	
>60	343	1.353 (0.934–1.961)	0.110
Weight	527		
< = 80	242	Reference	
>80	285	1.035 (0.727–1.473)	0.848
Height	522		
< = 160	246	Reference	
>160	276	1.016 (0.713–1.450)	0.929
BMI	518		
< = 30	211	Reference	
>30	307	1.046 (0.730–1.500)	0.805
Histologic grade	540		
G1	98	Reference	
G2	120	2.156 (1.015–4.580)	0.046
G3	322	3.281 (1.708–6.300)	<0.001
Radiation therapy	527		
No	279	Reference	
Yes	248	1.095 (0.771–1.556)	0.613
TMEM41A	551		
Low	276	Reference	
High	275	1.974 (1.374–2.836)	<0.001

EC, endometrial cancer; PFI, progression-free interval.

### 3.5. *TMEM41A* overexpression associates with the EC immune microenvironment

**Fig 9:**
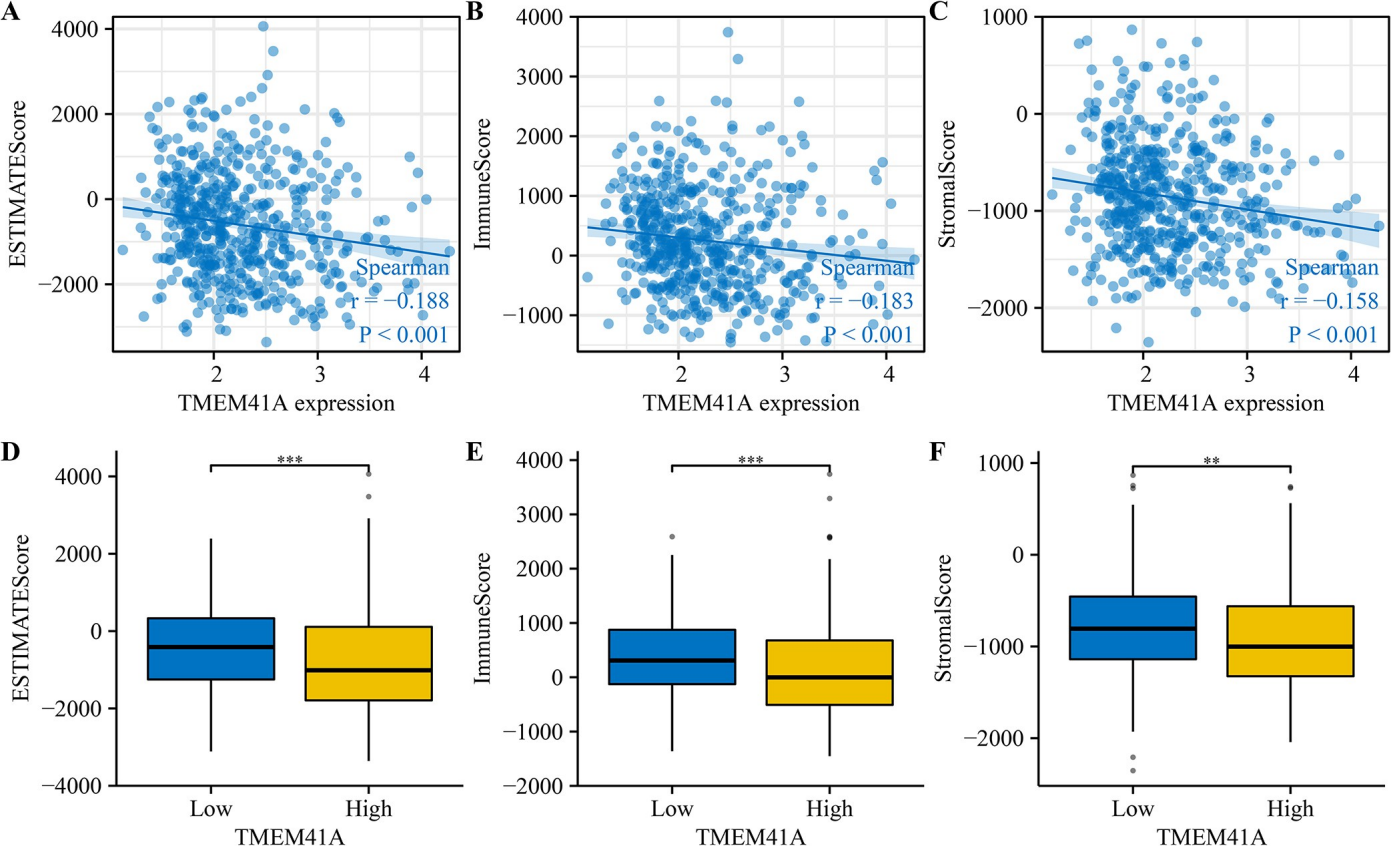
*TMEM41A* expression correlates with the EC stromal, immune, and estimate scores. (A-C) Correlation analysis. (D-F) Expression analysis. EC, endometrial cancer.

**Fig 10:**
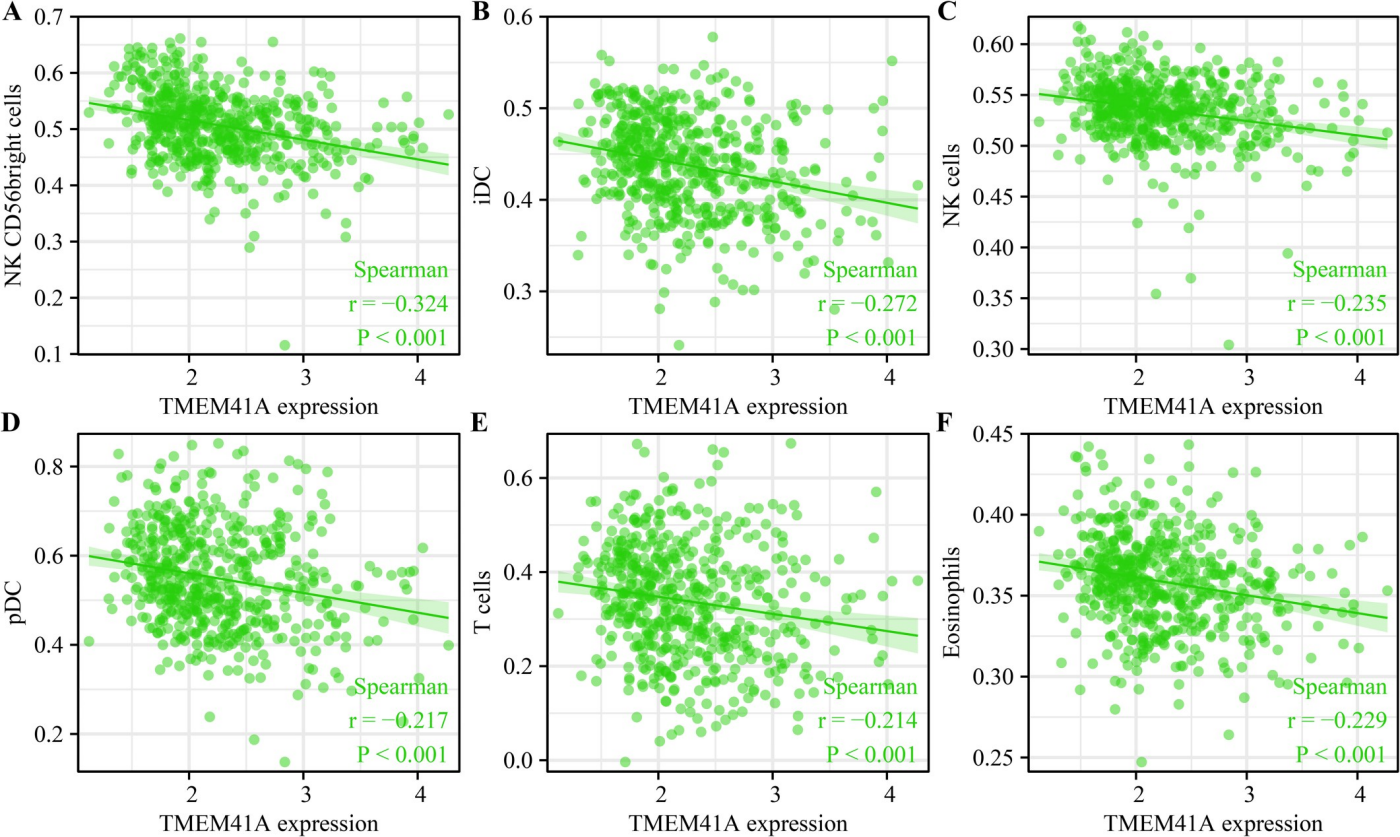
*TMEM41A* expression correlates with the EC immune cells. (A-F) Correlation analysis of *TMEM41A* expression with various immune cell types, including NK CD56 bright cells (A), iDC (B), NK cells (C), pDC (D), T cells (E), and Eosinophils (F). EC, endometrial cancer.

**Fig 11:**
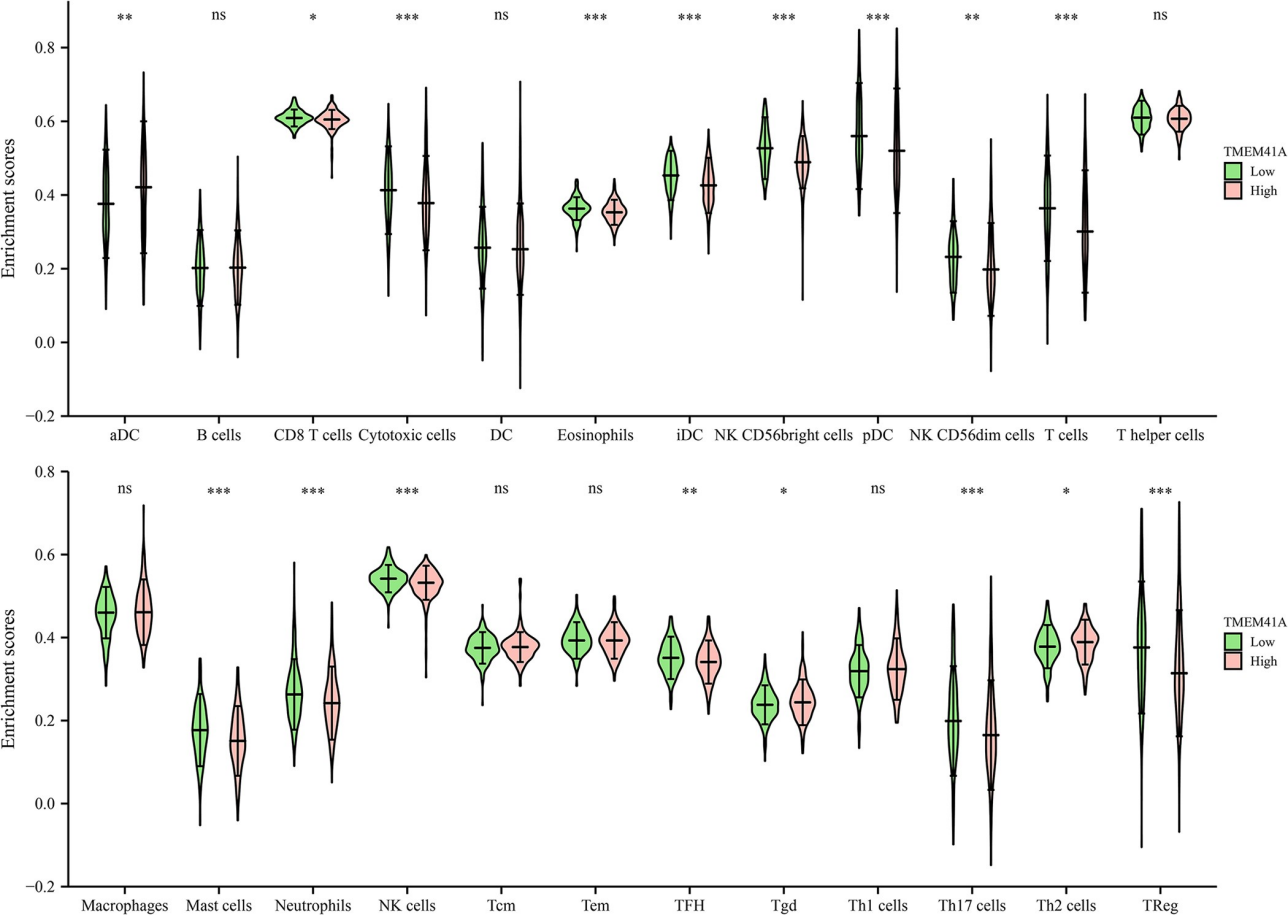
The expression levels of 24 immune cells in high- and low-*TMEM41A* expression groups.

**Fig 12:**
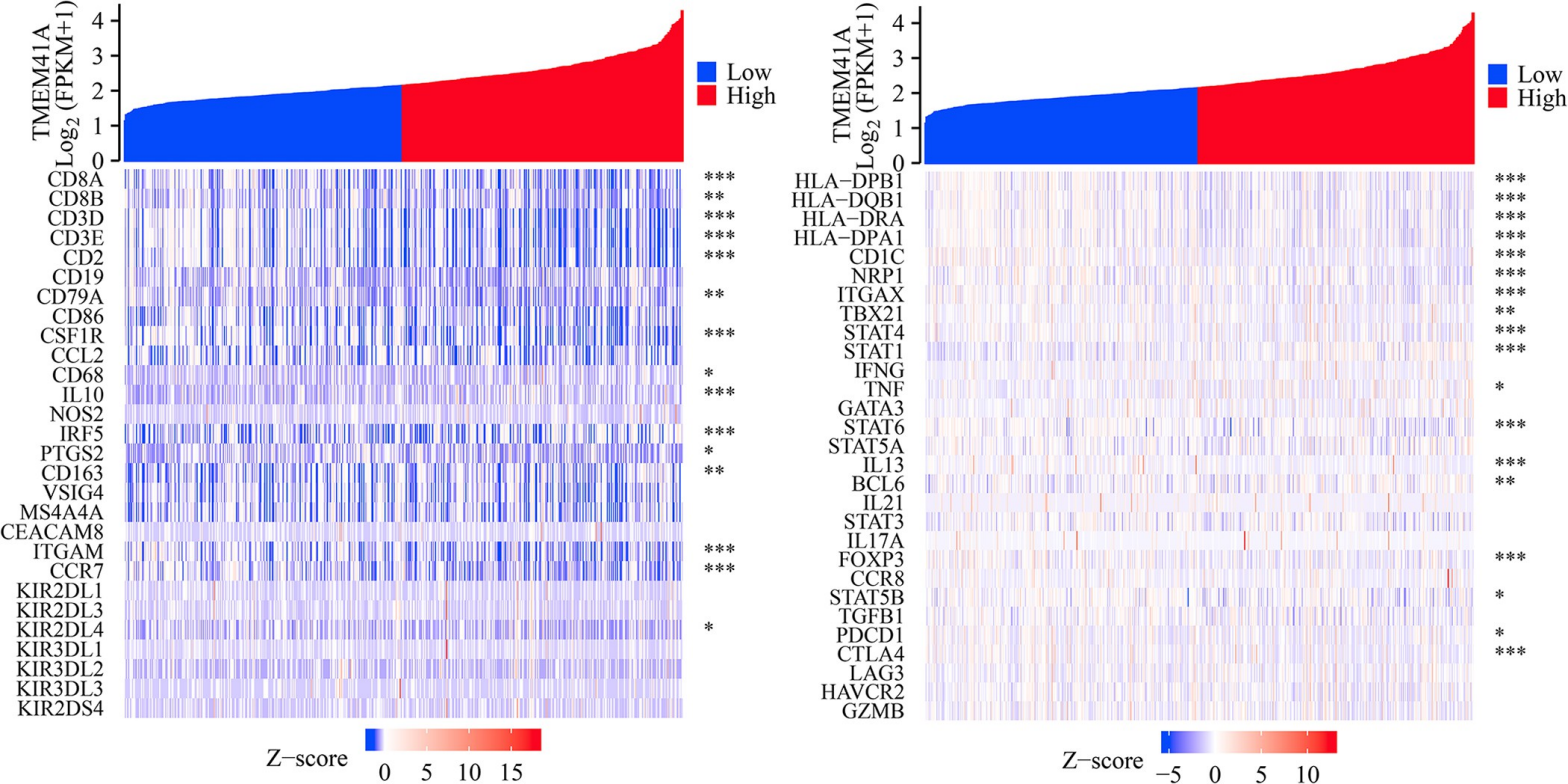
*TMEM41A* expression correlates with the immune cell markers.

*TMEM41A* overexpression was associated with stromal, immune, and estimate scores of cancer samples (Fig 9A–9C). Furthermore, stromal, immune, and estimate scores significantly differed between the high- and low-*TMEM41A* expression groups (Fig 9D–9F). In EC tissues of the TCGA database, *TMEM41A* overexpression correlated with the levels of macrophages, CD8^+^ T cells, TFH, Th2 cells, aDC, cytotoxic cells, eosinophils, iDC, mast cells, neutrophils, NK CD56bright cells, NK CD56dim cells, NK cells, pDC, T cells, Th17 cells, and TReg (Fig 10 and Table 5). The expression levels of 24 immune cell types in the high- and low*-TMEM41A* expression groups are presented in Fig 11. Additionally, we found that *TMEM41A* overexpression correlated with tumor purity, CD8^+^ T cells, macrophage, and neutrophil levels (S1 Fig), and with the copy numbers of CD8^+^ T cell, neutrophil, and DC in the TIMER database (S2 Fig). Finally, *TMEM41A* overexpression was also significantly associated with the levels of immune cell markers, including *CD8A*, *CD3D*, *CD3E*, *CD2*, *CSF1R*, *IL10*, *IRF5*, *ITGAM*, and *CCR7* (Fig 12).

**Table 5:** Overexpression of *TMEM41A* associates with the EC immune cells.

Target molecule	Immune cells	Correlation coefficient	P
TMEM41A	aDC	0.160	<0.001
TMEM41A	B cells	0.030	0.475
TMEM41A	CD8 T cells	-0.110	0.009
TMEM41A	Cytotoxic cells	-0.186	<0.001
TMEM41A	DC	-0.001	0.983
TMEM41A	Eosinophils	-0.229	<0.001
TMEM41A	iDC	-0.272	<0.001
TMEM41A	Macrophages	0.099	0.021
TMEM41A	Mast cells	-0.185	<0.001
TMEM41A	Neutrophils	-0.164	<0.001
TMEM41A	NK CD56bright cells	-0.324	<0.001
TMEM41A	NK CD56dim cells	-0.144	<0.001
TMEM41A	NK cells	-0.235	<0.001
TMEM41A	pDC	-0.217	<0.001
TMEM41A	T cells	-0.214	<0.001
TMEM41A	T helper cells	-0.040	0.351
TMEM41A	Tcm	0.033	0.445
TMEM41A	Tem	-0.057	0.181
TMEM41A	TFH	-0.133	0.002
TMEM41A	Tgd	0.067	0.118
TMEM41A	Th1 cells	0.032	0.455
TMEM41A	Th17 cells	-0.178	<0.001
TMEM41A	Th2 cells	0.131	0.002
TMEM41A	TReg	-0.208	<0.001

EC, endometrial cancer.

### 3.6. *TMEM41A* expression correlates with the RNA modification

In the RM2Target database, *ALKBH3*, *ALKBH5*, *ALYREF*, *ELAVL1*, *FMR1*, *FTO*, *HNRNPC*, *HNRNPA2B1*, *IGF2BP1*, *IGF2BP3*, *METTL1*, *METTL14*, *METTL16*, *METTL3*, *METTL5*, *PCIF1*, *RBMX*, *VIRMA*, *WDR4*, *WTAP*, *YBX1*, *YTHDC1*, *YTHDF1*, *YTHDF2*, and *YTHDF3* were found to be involved in regulating *TMEM41A*. Additionally, we found a significant correlation between the expression levels of *TMEM41A* and *ALKBH3*, *ALYREF*, *FMR1*, *FTO*, *HNRNPC*, *IGF2BP1*, *IGF2BP3*, *METTL1*, *METTL5*, *PCIF1*, *RBMX*, *VIRMA*, *WTAP*, *YBX1*, *YTHDF1*, *YTHDF2*, *YTHDF3*, *YTHDC1*, *ALKBH5*, *HNRNPA2B1*, and others using Spearman analysis (Table 6).

**Table 6:** *TMEM41A* expression correlates with the gene levels of RNA modification.

Target molecule	Gene	Correlation coefficient	P
TMEM41A	METTL3	0.001	0.983
TMEM41A	METTL16	-0.017	0.692
TMEM41A	ELAVL1	0.034	0.419
TMEM41A	WDR4	0.057	0.178
TMEM41A	METTL14	0.072	0.092
TMEM41A	YTHDC1	0.105	0.013
TMEM41A	ALKBH5	-0.117	0.006
TMEM41A	HNRNPA2B1	0.116	0.006
TMEM41A	ALKBH3	0.301	<0.001
TMEM41A	ALYREF	0.256	<0.001
TMEM41A	FMR1	0.335	<0.001
TMEM41A	FTO	-0.221	<0.001
TMEM41A	HNRNPC	0.155	<0.001
TMEM41A	IGF2BP1	0.463	<0.001
TMEM41A	IGF2BP3	0.267	<0.001
TMEM41A	METTL1	0.215	<0.001
TMEM41A	METTL5	0.295	<0.001
TMEM41A	PCIF1	0.221	<0.001
TMEM41A	RBMX	0.198	<0.001
TMEM41A	VIRMA	0.384	<0.001
TMEM41A	WTAP	0.283	<0.001
TMEM41A	YBX1	0.232	<0.001
TMEM41A	YTHDF1	0.342	<0.001
TMEM41A	YTHDF2	0.143	<0.001
TMEM41A	YTHDF3	0.248	<0.001

## 4. Discussion

In recent years, there has been growing interest in identifying new biomarkers for EC. Several studies have focused on identifying and characterizing the roles of novel oncogenes in EC progression and prognosis [14–17]. For instance, OTU deubiquitinase, ubiquitin aldehyde binding 2 (*OTUB2*) expression was significantly enhanced in EC, which was associated with poor prognosis of cancer patients. Overexpression of *OTUB2* could promote glycolysis and induce proliferation, migration, and invasion of EC cells, while inhibition of the PKM2/PI3K/AKT signaling pathway significantly reversed the oncogenic effects of OTUB2 overexpression on EC cells [15]. Similarly, ubiquitin-specific peptidase 5 (*USP5*) was significantly upregulated in EC tissues, whereas its inhibition could reduce the migration and proliferation ability of EC cells and induce cell cycle arrest and apoptosis. These effects of *USP5* overexpression were associated with excessive activation of the mTOR/4EBP1 signaling pathway [16]. These results suggested that targeting these biomarkers could offer potential therapeutic benefits for patients with EC. *TMEM41A* is a newly discovered oncogene [6, 7]. Studies have shown that the *SPRING1* could enhance colorectal cancer cell growth by promoting *TMEM41A* expression [6]. *TMEM41A* is also associated with lymph node metastasis, distant metastasis, and poor prognosis in patients with gastric cancer. Inhibition of *TMEM41A* expression could delay gastric cancer cell metastasis by regulating EMT and autophagy [7]. In this study, we found that *TMEM41A* was overexpressed in EC and was a potential diagnostic and prognostic marker for the disease. Our comprehensive analysis revealed that *TMEM41A* overexpression was associated with shorter OS, clinical stage, age, DSS, weight, histological subtype, PFI, tumor grade, race, and menopausal status in patients with EC. Our preliminary findings indicated that *TMEM41A* played an oncogenic role in EC, which is consistent with previous reports, and indicates that *TMEM41A* could be a valuable biomarker for predicting EC prognosis and guiding personalized treatment strategies.

Nomograms have emerged as an increasingly popular method for predicting the prognosis of patients with cancer [18–20]. For example, Wang et al. constructed a nomogram consisting of histological subtype, tumor grade, depth of invasion, cervical involvement, parametrial involvement, and HGB level to accurately predict the risk of lymph node metastasis in patients with EC [19]. Similarly, Li and Yue identified age, race, marital status, FIGO stage, grade, and metastasis as important prognostic factors using univariate Cox regression analysis. The risk factor-related nomogram had predictive power and was associated with the prognosis of patients with EC [20]. In this study, we identified clinical stage, age, histological grade, radiotherapy, and *TMEM41A* overexpression as risk factors for shorter OS in patients with EC. Moreover, clinical stage and *TMEM41A* overexpression were risk factors for poor DSS in patients with EC, while clinical stage, histological grade, and *TMEM41A* overexpression were risk factors for shorter PFI in patients with EC. The risk factors-related prognostic nomograms predicted the survival time of patients with EC, providing novel tools for evaluating the prognosis of patients with endometrial cancer.

Immunotherapy has emerged as a promising strategy to improve the prognosis of cancer patients [21–23]. For example, patients with esophageal squamous cell carcinoma treated with an anti-programmed death 1 (*PD-1*) inhibitor plus chemotherapy reported a median OS of 15.3 months and a median progression-free survival (PFS) of 6.9 months, compared with 12.0 months and 5.6 months in the chemotherapy group. This study demonstrated that adding anti-*PD-1* therapy to chemotherapy significantly improves survival in patients with advanced or metastatic esophageal squamous cell carcinoma [22]. Therefore, we explored the association between *TMEM41A* expression and components of the immune microenvironment in EC. We found that *TMEM41A* overexpression was correlated with EC stromal, immune, and estimate scores and was linked with tumor purity, immune cells (such as the macrophages, CD8 T cells, and TFH), and immune cell markers (such as the CD8A, CD3D, and CD3E). In addition, *TMEM41A* expression was significantly correlated with the levels of several RNA modification genes, including *ALKBH3*, *ALYREF*, *FMR1*, *FTO*, *HNRNPC*, *IGF2BP1*, *IGF2BP3*, *METTL1*, *METTL5*, *PCIF1*, *RBMX*, *VIRMA*, *WTAP*, *YBX1*, *YTHDF1*, *YTHDF2*, *YTHDF3*, *YTHDC1*, *ALKBH5*, and *HNRNPA2B1*. RNA modification genes play important regulatory roles in cancer [24–27]. For instance, *FTO*, the m6A modification gene, was highly expressed in metastatic EC tissues, and promoted EC metastasis and invasion by activating the Wnt signaling pathway through the demethylation of *HOXB13* mRNA, which eliminated recognition of m6A modification via the *YTHDF2* protein [27]. Additionally, the expression level of lncRNA FENDRR was decreased in endometrioid endometrial carcinoma (EEC), while the FENDRR m6A methylation level was significantly increased. *YTHDF2* mediated the degradation of FENDRR and promoted cancer cell proliferation by increasing *SOX4* expression in EEC [23]. Our findings demonstrated that *TMEM41A* was associated with immune microenvironment and RNA modification, further suggesting that *TMEM41A* may play a critical role in EC.

Traditionally, local invasion and histological characteristics have been used as risk factors for the prognosis of patients with EC. However, in recent years, molecular and genomic maps have emerged as promising tools to predict the prognosis of patients with EC, helping in identifying those with low, medium, and high recurrence risks [17, 28–30]. Our results revealed that *TMEM41A* was upregulated in EC and that its increased expression was related to poor prognosis, immune status, and RNA modification in patients with EC. This study provides new biomarkers for evaluating the prognosis of patients with EC and has the following advantages and disadvantages. We analyzed a large number of tissue samples and prognostic data, ensuring the reliability of our findings. In addition, this study is the first to report the association between *TMEM41A* expression and EC progression, providing new directions for future research. However, this study lacked basic research to confirm the current results. Our future studies would focus on collecting cancer tissues and normal adjacent tissues from patients with EC to verify the expression of *TMEM41A* and explore its impact on the prognosis of patients with EC. Moreover, the effects of inhibiting *TMEM41A* on the growth and migration of EC cells should be investigated. Additionally, radiomic features are crucial in the screening, diagnosis, and prognosis of patients with EC [30]. Therefore, future studies should combine *TMEM41A* expression and radiomic features to evaluate the prognosis of patients with EC.

## 5. Conclusions

In conclusion, our study demonstrates that *TMEM41A* exhibits strong expression in EC tissues. *TMEM41A* could serve as a diagnostic marker and evaluate the poor prognosis of patients with EC. Moreover, the overexpression of *TMEM41A* is associated with poor prognosis, stromal score, immune score, estimate score, immune cells, cell markers, and RNA modifications. These findings suggest that TMEM41A may serve as a potential biomarker for EC treatment.

## Supporting information

S1 Fig *TMEM41A* expression correlates with EC immunity. EC, endometrial cancer.
(JPG)

S2 Fig *TMEM41A* expression correlates with the immune cell copy number in EC. EC, endometrial cancer.
(JPG)

## References

[1] Wang C, Yin Y, Sun Z, Wang Y, Li F, Wang Y, et al. ATAD2 Upregulation Promotes Tumor Growth and Angiogenesis in Endometrial Cancer and Is Associated with Its Immune Infiltration. Dis Markers. 2022; 2022: 2334338. doi: 10.1155/2022/2334338 . 36479043 PMC9722300

[2] Song Y, Zhou G, Song E, Zhan L, Wang Q, Song H, et al. TRIM44 Promotes Endometrial Carcinoma Progression by Activating the FRS2 Signalling Pathway. Evid Based Complement Alternat Med. 2022; 2022: 6235771. doi: 10.1155/2022/6235771 . 36387361 PMC9663230

[3] Post CCB, Westermann AM, Bosse T, Creutzberg CL, Kroep JR. PARP and PD-1/PD-L1 checkpoint inhibition in recurrent or metastatic endometrial cancer. Crit Rev Oncol Hematol. 2020; 152: 102973. doi: 10.1016/j.critrevonc.2020.102973 . 32497971

[4] Li D, Li K, Zhang W, Yang KW, Mu DA, Jiang GJ, et al. The m6A/m5C/m1A Regulated Gene Signature Predicts the Prognosis and Correlates With the Immune Status of Hepatocellular Carcinoma. Front Immunol. 2022; 13: 918140. doi: 10.3389/fimmu.2022.918140 . 35833147 PMC9272990

[5] Peng M, Fan S, Li J, Zhou X, Liao Q, Tang F, et al. Programmed death-ligand 1 signaling and expression are reversible by lycopene via PI3K/AKT and Raf/MEK/ERK pathways in tongue squamous cell carcinoma. Genes Nutr. 2022; 17(1): 3. doi: 10.1186/s12263-022-00705-y . 35164673 PMC8903509

[6] Tao Y, Luo J, Zhu H, Chu Y, Pei L. Chromosome 12 Open Reading Frame 49 Promotes Tumor Growth and Predicts Poor Prognosis in Colorectal Cancer. Dig Dis Sci. 2022; undefined: undefined. doi: 10.1007/s10620-022-07751-x . 36348128 PMC10102024

[7] Lin B, Xue Y, Qi C, Chen X, Mao W. Expression of transmembrane protein 41A is associated with metastasis via the modulation of E‑cadherin in radically resected gastric cancer. Mol Med Rep. 2018; 18(3): 2963–2972. doi: 10.3892/mmr.2018.9241 . 30015937

[8] Guo Q, Liu XL, Liu HS, Luo XY, Yuan Y, Ji YM, et al. The Risk Model Based on the Three Oxidative Stress-Related Genes Evaluates the Prognosis of LAC Patients. Oxid Med Cell Longev. 2022; 2022: 4022896. doi: 10.1155/2022/4022896 ; PMCID. 35783192 PMC9246616

[9] Lin Z, Huang L, Li S, Gu J, Cui X, Zhou Y. Pan-cancer analysis of genomic properties and clinical outcome associated with tumor tertiary lymphoid structure. Sci Rep. 2020; 10(1): 21530. doi: 10.1038/s41598-020-78560-3 . 33299035 PMC7725838

[10] Zhao YY, Xiang QM, Chen JL, Zhang L, Zheng WL, et al. SLC25A25-AS1 over-expression could be predicted the dismal prognosis and was related to the immune microenvironment in prostate cancer. Front Oncol. 2022; 12: 990247. doi: 10.3389/fonc.2022.990247 . 36338724 PMC9632290

[11] Xu Q, Ge Q, Zhou Y, Yang B, Yang Q, Jiang S, et al. MELK promotes Endometrial carcinoma progression via activating mTOR signaling pathway. EBioMedicine. 2020; 51: 102609. doi: 10.1016/j.ebiom.2019.102609 Epub 2020 Jan 6. . 31915116 PMC7000338

[12] Guo Q, Wu CY, Jiang N, Tong S, Wan JH, Xiao XY, et al. Downregulation of T-cell cytotoxic marker IL18R1 promotes cancer proliferation and migration and is associated with dismal prognosis and immunity in lung squamous cell carcinoma. Front Immunol. 2022; 13: 986447. doi: 10.3389/fimmu.2022.986447 . 36544782 PMC9760870

[13] Li Y, Huo J, He J, Zhang Y, Ma X. BTG1 inhibits malignancy as a novel prognosis signature in endometrial carcinoma. Cancer Cell Int. 2020; 20: 490. doi: 10.1186/s12935-020-01591-3 . 33041670 PMC7542768

[14] Hu A, Wang Y, Tian J, Chen Z, Chen R, Han X, et al. Pan-cancer analysis reveals DDX21 as a potential biomarker for the prognosis of multiple tumor types. Front Oncol. 2022; 12: 947054. doi: 10.3389/fonc.2022.947054 . 36505822 PMC9730287

[15] Zhang Q, Zhang J, Yao A, Tian X, Han Z, Yuan Y, et al. OTUB2 promotes the progression of endometrial cancer by regulating the PKM2-mediated PI3K/AKT signaling pathway. Cell Biol Int. 2023; 47(2):428–438. doi: 10.1002/cbin.11950 . 36316812

[16] Li Y, Zhou J. USP5 Promotes Uterine Corpus Endometrial Carcinoma Cell Growth and Migration via mTOR/4EBP1 Activation. Cancer Manag Res. 2021; 13: 3913–3924. doi: 10.2147/CMAR.S290467 . 34012297 PMC8128349

[17] Di Donato V, Giannini A, Bogani G. Recent Advances in Endometrial Cancer Management. J Clin Med. 2023;12(6): 2241. doi: 10.3390/jcm12062241 . 36983243 PMC10053513

[18] Wu J, Zhang H, Li L, Hu M, Chen L, Xu B, et al. A nomogram for predicting overall survival in patients with low-grade endometrial stromal sarcoma: A population-based analysis. Cancer Commun (Lond). 2020; 40(7): 301–312. doi: 10.1002/cac2.12067 . 32558385 PMC7365459

[19] Wang Z, Zhang S, Ma Y, Li W, Tian J, Liu T. A nomogram prediction model for lymph node metastasis in endometrial cancer patients. BMC Cancer. 2021; 21(1): 748. doi: 10.1186/s12885-021-08466-4 . 34187416 PMC8243766

[20] Li R, Yue Q. A nomogram for predicting overall survival in patients with endometrial carcinoma: A SEER-based study. Int J Gynaecol Obstet. 2022; undefined: undefined. doi: 10.1002/ijgo.14580 . 36394432

[21] Doroshow DB, Bhalla S, Beasley MB, Sholl LM, Kerr KM, Gnjatic S, et al. PD-L1 as a biomarker of response to immune-checkpoint inhibitors. Nat Rev Clin Oncol. 2021; 18(6): 345–362. doi: 10.1038/s41571-021-00473-5 . 33580222

[22] Luo H, Lu J, Bai Y, Mao T, Wang J, Fan Q, et al. Effect of Camrelizumab vs Placebo Added to Chemotherapy on Survival and Progression-Free Survival in Patients With Advanced or Metastatic Esophageal Squamous Cell Carcinoma: The ESCORT-1st Randomized Clinical Trial. JAMA. 2021; 326(10): 916–925. doi: 10.1001/jama.2021.12836 . 34519801 PMC8441593

[23] Tawbi HA, Schadendorf D, Lipson EJ, Ascierto PA, Matamala L, Castillo Gutiérrez E, et al. Relatlimab and Nivolumab versus Nivolumab in Untreated Advanced Melanoma. N Engl J Med. 2022; 386(1): 24–34. doi: 10.1056/NEJMoa2109970 . 34986285 PMC9844513

[24] Shen J, Feng XP, Hu RB, Wang H, Wang YL, Qian JH, et al. N-methyladenosine reader YTHDF2-mediated long noncoding RNA FENDRR degradation promotes cell proliferation in endometrioid endometrial carcinoma. Lab Invest. 2021; 101(6): 775–784. doi: 10.1038/s41374-021-00543-3 . 33692441

[25] Li Q, Wang C, Dong W, Su Y, Ma Z. WTAP facilitates progression of endometrial cancer via CAV-1/NF-κB axis. Cell Biol Int. 2021; 45(6): 1269–1277. doi: 10.1002/cbin.11570 . 33559954

[26] Zhang L, Wan Y, Zhang Z, Jiang Y, Gu Z, Ma X, et al. IGF2BP1 overexpression stabilizes PEG10 mRNA in an m6A-dependent manner and promotes endometrial cancer progression. Theranostics. 2021; 11(3): 1100–1114. doi: 10.7150/thno.49345 . 33391523 PMC7738899

[27] Zhang L, Wan Y, Zhang Z, Jiang Y, Lang J, Cheng W, et al. FTO demethylates m6A modifications in HOXB13 mRNA and promotes endometrial cancer metastasis by activating the WNT signalling pathway. RNA Biol. 2021; 18(9): 1265–1278. doi: 10.1080/15476286.2020.1841458 . 33103587 PMC8354663

[28] Cuccu I, D’Oria O, Sgamba L, De Angelis E, Golia D’Augè T, Turetta C, et al. Role of Genomic and Molecular Biology in the Modulation of the Treatment of Endometrial Cancer: Narrative Review and Perspectives. Healthcare (Basel). 2023; 11(4): 571. doi: 10.3390/healthcare11040571 . 36833105 PMC9957190

[29] Golia D’Augè T, Cuccu I, Santangelo G, Muzii L, Giannini A, Bogani G, et al. Novel Insights into Molecular Mechanisms of Endometrial Diseases. Biomolecules. 2023; 13(3): 499. doi: 10.3390/biom13030499 . 36979434 PMC10046407

[30] Bogani G, Chiappa V, Lopez S, Salvatore C, Interlenghi M, D’Oria O, et al. Radiomics and Molecular Classification in Endometrial Cancer (The ROME Study): A Step Forward to a Simplified Precision Medicine. Healthcare (Basel). 2022; 10(12): 2464. doi: 10.3390/healthcare10122464 . 36553988 PMC9778151

