# Expression of Concern: TMEM41A overexpression correlates with poor prognosis and immune alterations in patients with endometrial carcinoma

**DOI:** 10.1371/journal.pone.0348229

**Published:** 2026-04-29

**Authors:** The PLOS One Editors

The *PLOS One* Editors issue this Expression of Concern because this article [1] was identified as one of a series of submissions for which we have concerns about the peer review process. Readers are advised to interpret the article [1] with caution.

The Editors have no evidence of any author involvement in the peer review concerns.

Additionally, after this article [1] was published, the following concerns were noted:

- The article cited as Reference 2 was retracted after [1] was accepted for publication and before [1] was published.
- The article cited as Reference 8 was retracted after [1] was published.
