# Supplementary figures and images for: TMEM41A overexpression correlates with poor prognosis and immune alterations in patients with endometrial carcinoma
Source: PLoS One. 2023 Jul 21;18(7):e0285817. doi: 10.1371/journal.pone.0285817 (PMC10361503; doi:10.1371/journal.pone.0285817)

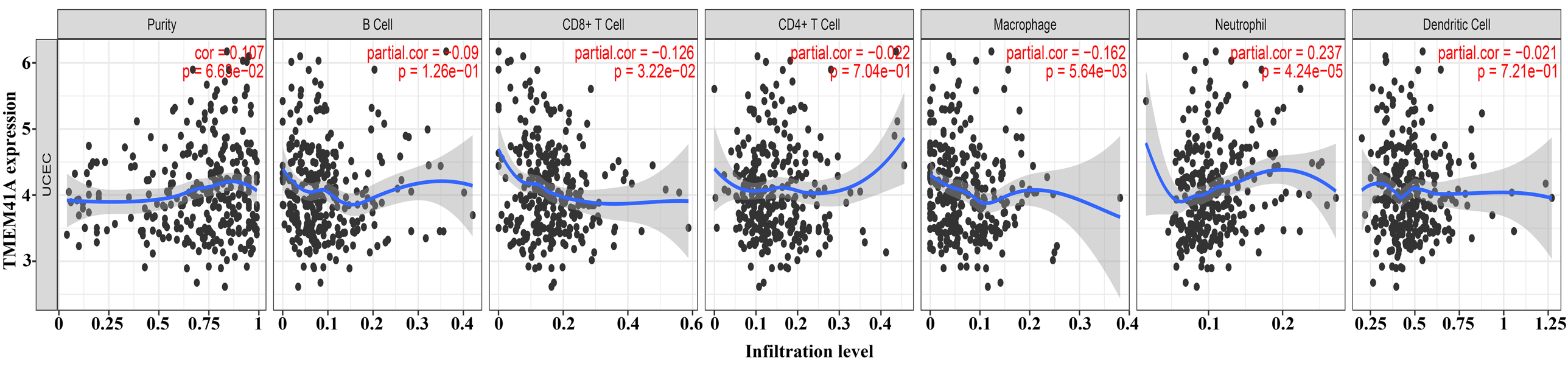

Supplement: S1 Fig — EC, endometrial cancer. (JPG) [file pone.0285817.s001.jpg]

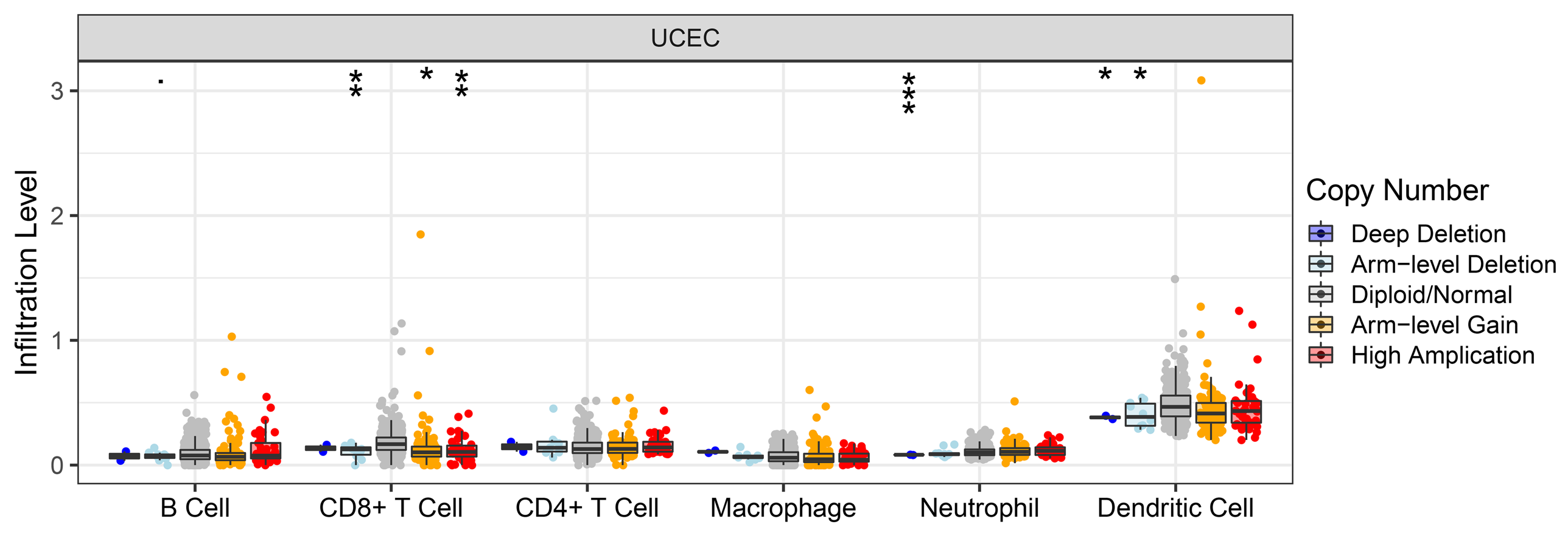

Supplement: S2 Fig — EC, endometrial cancer. (JPG) [file pone.0285817.s002.jpg]
